# A technology-enabled Counselling program versus a delayed treatment control to support physical activity participation in people with inflammatory arthritis: study protocol for the OPAM-IA randomized controlled trial

**DOI:** 10.1186/s41927-017-0005-4

**Published:** 2017-11-28

**Authors:** Linda C. Li, Lynne M. Feehan, Chris Shaw, Hui Xie, Eric C. Sayre, Antonio Aviña-Zubeita, Navi Grewal, Anne F. Townsend, Diane Gromala, Greg Noonan, Catherine L. Backman

**Affiliations:** 1 Department of Physical Therapy, University of British Columbia, Friedman Building, 2177 Wesbrook Mall, Vancouver, BC Canada; 2 Arthritis Research Canada, Milan Ilich Arthritis Research Centre, 5591 No. 3 Road, Richmond, BC V6X 2C7 Canada; 3 School of Interactive Arts and Technology, Simon Fraser University, 250-13450 102 Avenue, Surrey, BC V3T 0A3 Canada; 4 Faculty of Health Sciences, Simon Fraser University, 8888 University Drive, Burnaby, BC Canada; 5 Department of Medicine, University of British Columbia, 2775 Laurel Street, 10th Floor, Vancouver, BC V5Z 1M9 Canada; 6 Medical School, University of Exeter, St Luke’s Campus, Heavitree Road, Exeter, EX1 2LU UK; 7 Mary Pack Arthritis Program, Vancouver General Hospital, 895 W 10th Avenue, Vancouver, BC Canada; 8 Department of Occupational Therapy and Occupational Science, University of British Columbia, 2211 Wesbrook Mall T325, Vancouver, BC V6T 2B5 Canada

**Keywords:** Physical activity, Arthritis, Wearables, Behavioural interventions, Counselling

## Abstract

**Background:**

Being physically active is an essential component of successful self-management for people with inflammatory arthritis; however, the vast majority of patients are inactive. This study aims to determine whether a technology-enabled counselling intervention can improve physical activity participation and patient outcomes.

**Methods:**

The Effectiveness of Online Physical Activity Monitoring in Inflammatory Arthritis (OPAM-IA) project is a community-based randomized controlled trial with a delayed control design. We will recruit 130 people with rheumatoid arthritis or systemic lupus erythematosus, who can be physically active without health professional supervision. Randomization will be stratified by diagnosis. In Weeks 1–8, participants in the Immediate Group will: 1) receive education and counselling by a physical therapist (PT), 2) use a Fitbit and a new web-based application, FitViz, to track and obtain feedback about their physical activity, 3) receive 4 biweekly follow-up calls from the PT. Those in the Delayed Group will receive the same program in Week 10. We will interview a sample of participants about their experiences with the intervention. Participants will be assessed at baseline, and Weeks 9, 18 and 27. The primary outcome measure is time spent in moderate/vigorous physical activity in bouts of ≥ 10 min, measured with a portable multi-sensor device in the free-living environment. Secondary outcomes include step count, time in sedentary behaviour, pain, fatigue, mood, self-management capacity, and habitual behaviour.

**Discussion:**

A limitation of this study is that participants, who also administer the outcome measures, will not be blinded. Nonetheless, by customizing existing self-monitoring technologies in a patient-centred manner, individuals can be coached to engage in an active lifestyle and monitor their performance. The results will determine if this intervention improves physical activity participation. The qualitative interviews will also provide insight into a paradigm to integrate this program to support self-management.

**Trial registration:**

Date of last update in ClinicalTrials.gov: September 18, 2015. ClinicalTrials.gov Identifier: NCT02554474.

**Electronic supplementary material:**

The online version of this article (10.1186/s41927-017-0005-4) contains supplementary material, which is available to authorized users.

## Background

Arthritis is the most common cause of severe chronic pain and disability worldwide [1, 2]. Promoting physical activity is a priority because it is an essential adjunct to medical treatment for people with inflammatory arthritis (e.g., rheumatoid arthritis [RA]; systemic lupus erythematosus [SLE]) [3, 4], partly due to its effects in reducing risks of cardiovascular conditions and metabolic syndromes [5–8]. Physical activity level is also inversely associated with inflammatory markers such as C-reactive protein level and erythrocyte sedimentation rate in people with RA [6–8]. For people with SLE, physical activity is important due to its positive effect on sleep quality [9] and fatigue [10, 11], which is the most prevalent and debilitating symptom [12].

Public health guidelines recommend at least 150 min a week of moderate/vigorous physical activity (MVPA), performed in bouts of 10 or more minutes [13]. However, the majority of people with arthritis do not meet the recommendations. The Canadian Community Health Survey reported that over 57% of people with arthritis were physically inactive during their leisure time, compared to 46% of those without arthritis [14]. A 2012 study using accelerometers found 42% of those with RA [15] accumulated 0 min (in bouts) of MVPA in the preceding 7 days. The poor level of participation in physical activity in these populations represent a major knowledge-to-action gap.

Several modifiable risk factors are associated with low physical activity participation in people with arthritis. These include lack of motivation [16], doubts about the effectiveness of exercise [17], and lack of health professional advice [18]. Once patients start being active, they need feedback on their progress. A Cochrane review reported that ‘graded exercise activity’, which initially focuses on simple activities and then gradually increase to more challenging ones, is effective for improving adherence in people with chronic musculoskeletal condition [19]. Progression of activities can be guided by a physical therapist (PT) [19]. However, this is challenging to implement because only some parts of Canada have access to publicly funded arthritis-trained PTs for consultation [20]. The current knowledge on physical activity participation highlights the need for a new model of care that enables patients to monitor their activity performance, obtain feedback from health professionals and receive motivational support across geographic locations.

### Study aim and hypotheses

This study aims to determine whether a technology-enabled physical activity counselling intervention can improve physical activity participation in people with RA or SLE. We hypothesize that, compared to controls, those who receive the 8-week intervention will: 1) increase mean daily MVPA time as determined by an objective measure, 2) reduce mean daily sedentary time during waking hours, 3) have less pain, 4) have less fatigue, and 5) improve in perceived self-management capacity. In addition, we will explore the effect of the interventions on depressive symptoms and habitual behaviours.

### Study design

**Fig. 1:**
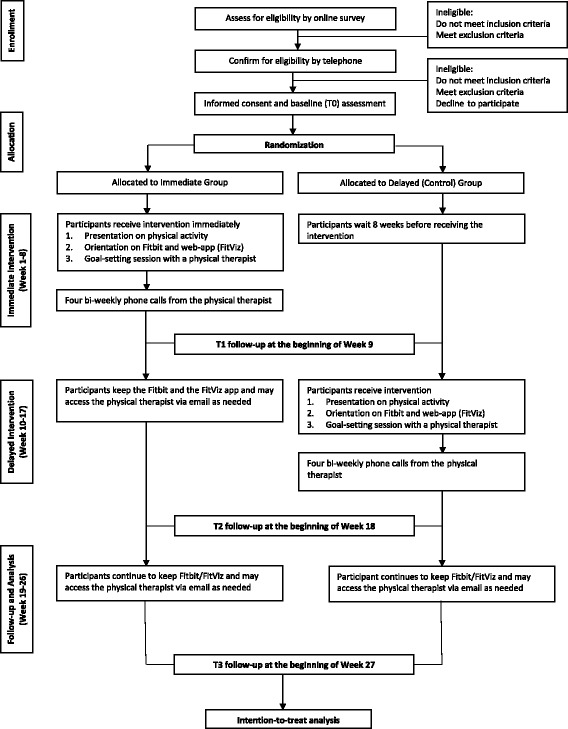
CONSORT flow diagram

The *Effectiveness of Online Physical Activity Monitoring in Inflammatory Arthritis (OPAM-IA)* project will employ a mix of quantitative and qualitative research methods. The intervention will be evaluated in a randomized controlled trial (RCT) with a delayed control design, whereby participants will be randomly assigned to start the intervention either immediately or at Week 10 (Fig. 1). This design is particularly suitable when the proposed intervention is likely to do more good than harm, as it allows all participants to receive the intervention by the end of the study. After completing the intervention, participants will partake in an in-depth interview by phone regarding their experiences. We have previously demonstrated feasibility of the study protocol in 34 people with osteoarthritis, with no dropout and 88% adhered to the protocol [21, 22]. In addition, we observed preliminary efficacy, with those who received the intervention showing a trend of improvement in MVPA and perceived self-management capacity compared to the controls after 1 month [22].

## Methods

### Participants

Eligible participants will be recruited from the Mary Pack Arthritis Program (Vancouver Coastal Health Authority) and Fraser Health Authority in British Columbia, Canada. Study information will also be posted on social media (Facebook, Twitter, Kajiji, Craigslist) and distributed by our patient pratner's organizations (Arthritis Research Canada, and Arthritis Consumer Experts). Individuals are eligible if they have a diagnosis of RA or SLE, have an email address and daily access to internet, and are able to attend the 1.5-h education session. We will exclude people who have previously used any physical activity wearables or are unsafe to be physically active without health professional supervision, as identified by the Physical Activity Readiness Questionnaire (PAR-Q) [23].

After completing the baseline measures, participants will be randomly assigned to the Immediate Group or the Delayed Group (i.e. control) in 1:1 allocation ratio. Randomization, stratified by diagnosis (RA or SLE), will be performed using numbers generated by SAS v9.4 (SAS Institute, Cary, North Carolina, USA) in variable block sizes to ensure adequate allocation concealment.

### Wearable and online technology

The intervention will include a Fitbit Flex 2™ wristband. Fitbit® is a commercial wearable device which tracks and displays steps walked, gross level of physical exertion, and the time spent being active. Fitbit® has an open source platform that permits customization of a new app, FitViz, to enhance the use of the data as part of our activity coaching strategy. To ensure user friendliness, FitViz was co-developed with 3 patient research partners from Arthritis Research Canada and Arthritis Consumer Experts. Using FitViz, the participant can share information with a study PT who will coach them to set activity goals by phone and adjust the activity parameters in the app remotely. These parameters include: 1) the upper and lower bound of intensity and duration of MVPA, 2) the duration when a sedentary behaviour should be interrupted, and 3) the rest time in between vigorous activities (i.e., pacing). By combining the use of a wearable, an app and coaching from a PT, we maximize the use of behavioural change techniques for supporting people with arthritis to engage in an active lifestyle [24].

### Intervention

The Immediate Group will receive the 8-week intervention immediately after randomization. Participants will attend a 1.5-h session where they receive: 1) 20 min of standardized education about physical activity, 2) a Fitbit Flex 2™ and a FitViz app account, and 3) individual coaching by a study PT trained in motivational interviewing [25]. The coaching will follow the Brief Action Planning approach [26], whereby the PT guides individuals to set goals, develop an action plan, and identify barriers and solutions. The PT will then adjust the activity parameters on the app based on the participants’ goals.

Participants’ physical activity will be captured continuously by the Fitbit® and wirelessly synchronized with FitViz 150 times/day. During Weeks 1–8, a study PT will review participants’ progress and coach them to modify their physical activity goals via 4 biweekly phone calls. A counselling guide will be used and the discussion will be documented by the PT. Participants may also contact the PT via email with questions. At the end of the intervention, participants may keep their Fitbit® and FitViz account, but will have no contact with a PT.

The Delayed Group will receive the intervention in Week 10. During the waiting period (Delayed Group only) or post-intervention period, participants will receive monthly emails of arthritis news, which are unrelated to physical activity.

To better understand the reasons people do or do not adopt and maintain recommended levels of physical activity, we will interview 20 participants with RA and 20 with SLE for 1 h by phone after the intervention. Interviews will focus on 1) goals set, strategies used, barriers/facilitators to being active, 2) their experience with the intervention, 3) the nature of activities they engage in, and 4) their experience of being a research participant. These data will enrich the RCT data, and inform the design of the future implementation strategy, if the intervention is found to be effective.

### Outcome measures

Participants will be assessed at baseline (T0), and Weeks 9 (T1), 18 (T2) and 27 (T3). Our primary outcome measure will be mean daily MVPA time measure with SenseWear Mini, a multi-sensor monitor that is worn on the upper arm over the triceps. It integrates tri-axial accelerometer data, physiological sensor data and personal demographic information to provide estimates of steps and energy expenditure. Tierney et al. [27] has showed that SenseWear is a valid tool for estimating energy expenditure during activities of daily living in people with RA (ICC = 0.72). A strong relationship was also found between SenseWear and indirect calorimetry measures of energy expenditure for activities of daily living (Pearson’s *r* = 0.85) [27]. SenseWear can be worn 24 h a day. Hence, it can capture a full picture of physical activity and the off-body time throughout the day [28, 29]. An important feature of SenseWear is its ability to differentiate between sedentary and light physical activities [30], making it an ideal instrument to assess both active and sedentary behaviours. Participants will wear a SenseWear Mini for 7 days at each assessment. Almeida et al. [31] determined that a minimum of 4 days of wear is required to reliably assess energy expenditure from different levels of physical activity in people with RA (ICC > .80).

We will calculate the average daily MVPA accumulated in bouts per day. A bout is defined as ≥ 10 consecutive minutes at the level of ≥ 3 METs (i.e., the lower bound of MVPA), with allowance for interruption of up to 2 min below the threshold [32]. Additional analysis will be performed with a cut-off at ≥ 4 METs which reflects purposeful activities [33].

Secondary outcomes will measure 1) mean daily time in sedentary behaviour, 2) average daily step count, 3) McGill Pain Questionnaire Short Form (MPQ-SF), 4) Fatigue Severity Scale, and 5) Partners in Health Scale. Sedentary behaviour and step count will be measured with SenseWear. For sedentary behaviour, we will calculate the mean daily time spent with an energy expenditure of ≤ 1.5 METs, occurring in bouts of > 20 min during waking hours [34–37]. The MPQ-SF contains 15 pain-related words, which can be rated from 0 to 3 (higher = more severe) [38]. The Fatigue Severity Scale, which consists of 9 questions measuring the impact of fatigue, has demonstrated excellent internal consistency (Cronbach’s α = 0.89) [12]. Construct validity was demonstrated by a moderate correlation with pain (*r* = 0.68) and depression (*r* = 0.46) [39]. The Partners in Health Scale is a 12-item measure designed to assess self-efficacy, knowledge of health conditions and treatment, and self-management behaviour such as adopting a healthy lifestyle (Cronbach’s α = 0.82) [40].

Tertiary outcome will include Patient Health Questionnaire-9 (PHQ-9) [41] and Self-Reported Habit Index [40]. The PHQ-9 consists of 9 questions (rated from 0 to 3) that correspond to the diagnostic criteria for major depressive disorder. A total score of greater than 11 indicates a major depressive disorder [41]. A difference of at least 5 points indicates clinical change over time [42]. The Self-Reported Habit Index is a 12-item scale, rated on a 7-point Likert scale, that measures characteristics of habitual behavior (reliability minimum α = 0.81). We will ask participants to rate their strength of habit for 3 specific activity-related behaviors: sitting during leisure time at home, sitting during usual occupational activities, and walking outside for 10 min. A higher score indicates a stronger habit or behaviour that is done frequently and automatically [43, 44].

### Data analysis and monitoring

#### Power calculation

Our collaboration with health authorities and patient groups will allow the study to recruit 130 eligible participants within 24 months. In one of our proof-of-concept studies on a similar physical activity counselling intervention involving 61 people with osteoarthritis, we estimated a standard deviation (SD) of 52.0 min of bouted MVPA performed in sessions of ≥ 10 min (unpublished data). Assuming an attrition rate of approximately 15%, we anticipate 110 of the 130 participants will complete the study (55 per group). With a sample size of 110 and α-level of 0.05, we will have 80.5% power to detect a between-group difference of at least 25 min post intervention (via one-sided test).

#### Intervention Fidelity and adverse event monitoring

We will monitor intervention fidelity by tracking participants’ Fitbit/FitViz app usage statistics (frequency & duration of use) during the evaluation periods. Further, we will analyze PTs’ physical activity counselling records to ensure the discussions follow the brief action planning approach. Participants will report any serious adverse events (falls, cardiovascular and musculoskeletal events) [45] to the study coordinator at any time during the study period. In addition, we will ask participants to record all adverse events related to their physical activity in the follow-up questionnaire at Weeks 9, 18 and 27.

#### Data analysis

An intention-to-treat analysis will be performed by a biostatistician who is blinded to the group assignment. For the main comparison, we will use the Shapiro-Wilk test to assess normality of the outcome variables. If normality assumption is rejected a suitable transformation will be selected to achieve an approximately normal distribution [46]. Analysis of covariance (ANCOVA) will be used to evaluate the effect of the intervention on the outcome measures, adjusting for 2 strata and blocking. If blocking is found to play no role, then it will be removed from the subsequent analyses.

Since we expect the randomization schedule to be implemented as planned, any differences between groups at baseline should be due to chance. Hence, the main analysis will not adjust for baseline differences [47, 48]. We will perform sensitivity analyses to adjust for baseline differences that appear to be clinically important to determine if they affect the conclusion from the main analysis. The first contrast will compare T0-T1 between the 2 groups to determine if the intervention is superior to the control. The second contrast will compare T0-T1 with T1-T2 in the Delayed Group. Unlike the first contrast which provides between-subjects treatment effect estimate, this second contrast uses within-subject pre-post comparison for treatment effect estimates. We will use linear mixed-effects longitudinal models to combine the first and second contrast for an overall treatment effect estimation. This combined estimate has the potential to substantially improve the precision of treatment effect estimates as compared with using either one alone. The third contrast will compare T0–T1 in the Immediate Group against T1-T2 in the Delayed Group. The forth contrast will compare T0-T1 in the Immediate Group against T1-T3 in the Delayed Group. The last two models will assess if the 10-week delay had an impact on the efficacy of the intervention. We will use descriptive analysis to summarize participant characteristics, comorbid conditions and adverse events, which will be adjudicated by the first author.

For the qualitative interviews, we will conduct an iterative content analysis, whereby codes will be identified and revised as interviews are analyzed. Initial open coding (i.e., assigning conceptual labels to the content) will be followed by clustering the labels into thematic categories. Quotes representative of the thematic categories will be identified to illustrate participants’ perspectives on physical activity, nature of activities, and their experiences as research participants. These data will inform the interpretation of statistical analyses and the design of future studies and implementation strategies, for example, ways for PTs to provide feedback about physical activity to people with inflammatory arthritis.

## Discussion

### Potential impact and significance of the study

More than 1 in 6 people in the U.S. are using wearable devices to monitor their health [49], but the integration of these tools in chronic disease management is still at an early stage. The *OPAM* project will evaluate a novel technology-enabled physical activity counselling intervention that adapts a popular wearable device to motivate and provide feedback to people with inflammatory arthritis regardless of their location. If shown to be effective, this intervention could inform a new person-centred approach to optimize self-management among people with arthritis. Furthermore, since being active is a key component of successful self-management, this intervention has potential to improve disease-related outcome and quality of life. Although RA and SLE are our current focus, the FitViz app is designed to be adaptable and scalable to serve people with other chronic diseases and to address other aspects of self-management (e.g., adding a self-report module to track medication use).

### Strengths and weaknesses of the study

This study has several strengths. Frist, we have previously demonstrated feasibility to deliver the remote counselling intervention to our target population with a high level of adherence to the protocol [22]. Second, our process to monitor participants’ Fitbit/FitViz use and the PT counselling will ensure intervention fidelity, which is important for maintaining internal validity of the study and enhancing external validity. Third, the rigorous mixed-methods design will enhance our ability to develop strategies to integrate this program in people’s daily life in the future. Finally, we anticipate that the pragmatic nature of the program will improve the chance of successful implementation in clinical practice, if it is shown to be effective.

A limitation of the study is that the intervention requires participants to use the device continuously for 8 weeks. To minimize non-compliance, we choose to use Fitbit Flex 2™ which can be worn on the wrist 24 h a day including during water-based activities. Our pilot study suggests that it is feasible for people with joint pain to use the device continuously for an extended period [22]. Also, it is possible that participants may gain access to a Fitbit during the non-intervention period since it is commercially available. To encourage adherence to the study protocol, participants may keep their device after the study period free of charge. FitViz will only be available to study participants through the study. We believe that these measures will minimize the risk of contamination in the RCT.

Supporting a physically active lifestyle is a core business of the physical therapy profession. The Exercise is Medicine initiative currently advocates for the creation and implementation of effective physical activity counselling strategies in treatment plans for patients around the world [50]. With the ubiquitous use of wearables and the popularity of the quantified-self movement [51], health professionals can now leverage the engaging power of technology to motivate, monitor and counsel patients living with chronic disease. PTs are in the position to lead in the effort to create, evaluate and integrate technology to improve physical activity participation of patients. To this end, the *OPAM* project will be a first step to generate the necessary evidence on this type of PT-led intervention to support patient self-management.

## Additional file

Additional file 1: Reviewer reports and AU response to reviewers. (DOCX 16 kb)

## Abbreviations

ANCOVA: Analysis of Covariance
ICC: Intraclass Correlation
MPQ-SF: McGill Pain Questionnaire Short Form
MVPA: Moderate-to-Vigorous Physical Activity
OPAM-IA: *Effectiveness of Online Physical Activity Monitoring in Inflammatory Arthritis Project*
PAR-Q: Physical Activity Readiness Questionnaire
PHQ-9: Patient Health Questionnaire-9
PT: Physical Therapist
RA: Rheumatoid Arthritis
RCT: Randomized Controlled Trial
SD: Standard Deviation
SLE: Systemic Lupus Erythematosus
