# Supplementary material for: A technology-enabled Counselling program versus a delayed treatment control to support physical activity participation in people with inflammatory arthritis: study protocol for the OPAM-IA randomized controlled trial
Source: BMC Rheumatol. 2017 Nov 28;1:6. doi: 10.1186/s41927-017-0005-4 (PMC6383592; doi:10.1186/s41927-017-0005-4)
Supplement: Supplementary file 1 — Reviewer reports and AU response to reviewers. (DOCX 16 kb) [file 41927_2017_5_MOESM1_ESM.docx]

**Open Peer Review summary**

Pre-publication versions of this article, reviewer reports and author comments to reviewers are available by contacting [info@biomedcentral.com](mailto:info@biomedcentral.com).

| Original submission |  |  |
| --- | --- | --- |
| 02 October 2017 | Submitted | Original manuscript |
| 09 October 2017 | Author responded | Author comments – Linda Li |
| Resubmission – Version 2 |  |  |
| 09 October 2017 | Submitted | Manuscript version 2 |
| Publishing |  |  |
| 19 October 2017 | Editorially accepted |  |
| 28 November 2017 | Article published | 10.1186/s41927-017-0005-4 |

### How does Open Peer Review work?

Open peer review is a system where authors know who the reviewers are, and the reviewers know who the authors are. If the manuscript is accepted, the named reviewer reports are published alongside the article. Pre-publication versions of the article are available by contacting [info@biomedcentral.com](mailto:%20mailto:info@biomedcentral.com).

You can find further information about the peer review system at <https://bmcrheumatol.biomedcentral.com/about#peer-review+policy>
